# Rsp activates expression of the Cnt system in *Staphylococcus aureus*

**DOI:** 10.1186/s12866-020-02013-0

**Published:** 2020-10-28

**Authors:** Laura Vinué, David C. Hooper

**Affiliations:** Division of Infectious Diseases, Massachusetts General Hospital, Harvard Medical School, 55 Fruit St, Boston, MA 02114-2696 USA

## Abstract

**Background:**

The Cnt system is crucial for the optimal import of essential metals in metal-limiting conditions and contributes to virulence in *S. aureus*. In a screen for regulators of efflux pumps in a phage-based ultra-high-density transposon library, we identified Rsp as a candidate regulator of the *cntE* gene.

**Results:**

A two-fold decrease in expression of all genes of the *cnt* operon was observed by RT-qPCR in the *rsp* mutant compared to the parental strain, indicating that Rsp acts as an activator of the *cnt* operon. To determine whether the Rsp activation depends on iron, we compared mutant and parent *cnt* expression under varying metal conditions. A 2-fold reduction in *cnt* gene expression was detected in the *rsp* mutant in TSB, and a slightly smaller decrease (1.9, 1.7, and 1.5-fold changes for *cntK*, *cmtA,* and *cntE* respectively) was observed after addition of dipyridyl. The greatest decrease was seen with addition of FeSO_4_ (4.1, 5.3 and 6.3-fold changes for *cntK*, *cmtA* and *cntE* respectively). These findings suggest that Rsp activates the *cnt* operon in low and high iron conditions. To study the relationship between Rsp and the *cnt* repressors Fur and Zur, we created single and double mutants. Both *fur* and *zur* single mutants had significant increases in *cnt* gene expression compared to the parental strain, as did the *fur rsp* double mutant. The *zur rsp* double mutant also had a significant increase in *cntK* expression and a trend in increases in *cntA* and *cntE* expression just below statistical significance. Thus, the ability of Fur and Zur to repress *cnt* gene expression are not eliminated by the presence of Rsp. However, there were significantly smaller increases in *cnt* gene expression in the double mutants compared to single mutants, suggesting that Rsp activation can still occur in the absence of these repressors. To determine if Rsp directly modulates expression of *cnt* genes, incubation of purified Rsp caused a DNA-specific band shift for the *cntK* and *cntA* promoters.

**Conclusions:**

Rsp activation may act to maintain basal cellular levels of staphylopine to scavenge free metals when needed, in addition to metal dependent regulation by Fur and Zur.

**Supplementary information:**

**Supplementary information** accompanies this paper at 10.1186/s12866-020-02013-0.

## Background

*Staphylococcus aureus* poses a serious risk to public health due to its prevalence as a colonizer of skin and nares, its ability to cause a wide range of infections, and the increasing incidence of antibiotic-resistant strains [1]. During infection, *S. aureus* is confronted with a robust host innate immune response and an environment with low availability of free iron, manganese, and zinc [1, 2]. To overcome this host defense mechanism, *S. aureus* has evolved a diverse array of metal acquisition strategies that facilitates its proliferation and pathogenesis in host tissues [2, 3]. *S. aureus* captures free metals from the host through high-affinity transporter systems or small chelating molecules called metallophores [3].

Staphylopine (StP) is a nicotianamine-like metallophore secreted by *S. aureus*, with a remarkably broad spectrum of metal ligands, including nickel, cobalt, copper, zinc, and iron [4, 5]. The nine-gene *cntKLMABCDFE* operon is required for StP synthesis and trafficking. The first three genes *cntK*-*M* are responsible for StP biosynthesis; *cntA-F* encode the importer of the StP-metal complex; and CntE is involved in the export of StP [4–7].

The Cnt system is repressed by Fur and Zur in the presence of iron and zinc, respectively, with tight control of the StP biosynthesis genes and a looser control of the genes responsible for its export and recovery [8]. Besides the iron-dependent regulator Fur, an iron-independent regulation of the *cnt* regulon has been considered but not yet identified [8].

In this study, we identified a novel regulator of the *cnt* operon that activates the system in addition to the metal-dependent Fur and Zur repressors.

## Results

### Regulation of the *cnt* operon by the AraC-type regulator, Rsp

Fur is an iron-dependent transcriptional regulator that uses Fe (II) as a cofactor and negatively regulates transcription of iron transport genes by binding to the two Fur boxes present in the *cnt* operon [8]. Besides the Fur-dependent regulation, a Fur-independent regulation by iron was also described, but these regulators have not been identified [8], and nothing is known about an iron-independent regulation of the *cnt* operon. In addition, no activators of the *cnt* system has been described.

In a screen for regulators of efflux pumps and/or exporters, using ciprofloxacin selection in a previously reported phage-based ultra-high-density transposon library procedure [9] we identified Rsp, as a candidate regulator of the *cntE* exporter gene. The screen used a selection at varying concentrations of ciprofloxacin, a substrate of both the NorA and NorB efflux pumps, and identified differences in knockouts of genes of transcriptional regulators.

**Fig. 1:**
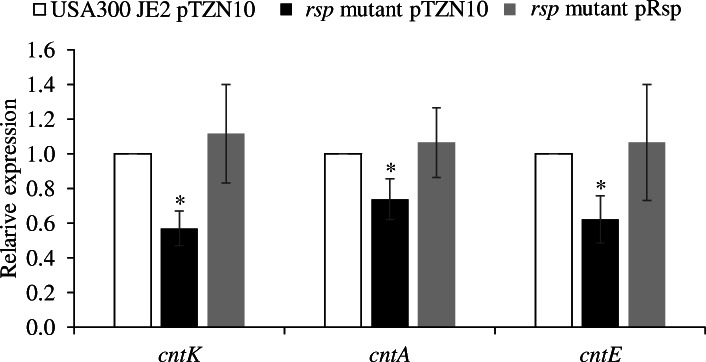
Complementation of Rsp in rich media. Relative expression of *cntK*, *cntA* and *cntE* genes in the *rsp* mutant strain with an empty plasmid pTZN10 and a plasmid expression *rsp* (pRsp) relative to USA300 JE2 with the empty plasmid were assessed by RT-qPCR. The bars represent the means of relative expression from at least three independent experiments. The error bars show standard errors of the means. Statistical differences were determined by Mann-Whitney U Test. *rsp* mutant pTZN10 showed lower expression for the three genes (*cntK, cntA, cntE*) than the USA300 JE2 pTZN10 strain (*P < 0.05), having no differences between USA300 JE2 pTZN10 and the complementation strain *rsp* mutant pRsp

To evaluate further the role of Rsp in *cnt* expression, a *rsp* mutant (NE1304) and its parental strain (USA300 JE2) from the Nebraska Transposon Mutant Library [10] were used to measure the expression of all genes of the *cnt* operon by RT-qPCR. As shown in Table 1, the expression of all genes in *cnt* operon decreased by an average of two-fold in the *rsp* mutant compared to the parental strain, being statistically significant for all genes of the operon, indicating that Rsp acts as an activator not only of *cntE* but also of the entire *cnt* operon. This finding was further supported by the results that restoration of the presence of Rsp via introducing plasmid encoding *rsp* gene (pRsp) reestablished the expression of *cntK*, *cntA* and *cntE* genes to the levels of wild type strain (USA300 JE2) with the empty plasmid as the control (Fig. 1).

**Table 1:** Relative expression of the *cnt* operon in a Δ*rsp* mutant strain

Genes	Fold change relative to USA300 JE2 (WT strain)	
	Mean	SEM
**StP biosynthesis**		
*cntK*	0.477	0.190
*cntL*	0.166	0.081
*cntM*	0.711	0.167
**StP importer**		
*cntA*	0.712	0.175
*cntB*	0.439	0.117
*cntC*	0.571	0.129
*cntD*	0.504	0.145
*cntF*	0.439	0.051
**StP exporter**		
*cntE*	0.377	0.052

*SEM* Error standard of the mean

### Rsp activation acts in iron-independent manner

**Fig. 2:**
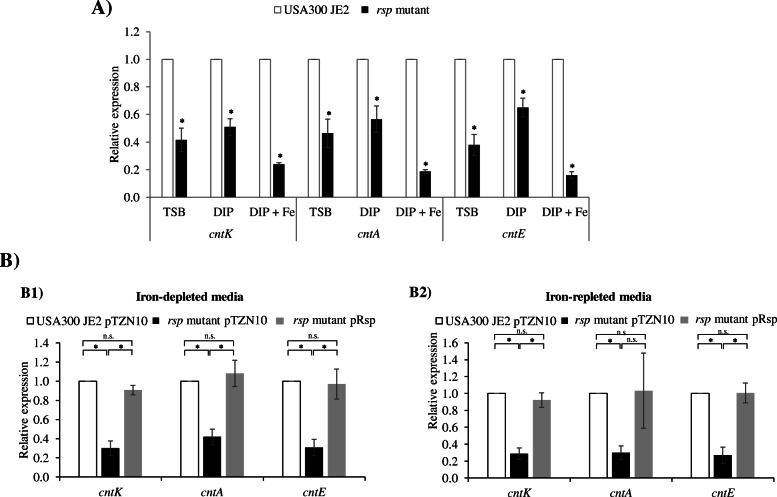
Rsp activation acts in iron-independent manner. **a** Relative expression of *cntK*, *cntA* and *cntE* genes in the *rsp* mutant strain relative to USA300 JE2 strain under different metal conditions: rich media (TSB), iron-depleted media (DIP = TSB + dipyridyl 400 μM) and iron-repleted media (DIP + 50 μM FeSO_4_) were assessed by RT-qPCR. **b** Complementation of Rsp. Relative expression of *cntK*, *cntA* and *cntE* genes in the *rsp* mutant strain with an empty plasmid pTZN10 and a plasmid expressing *rsp* (pRsp) relative to USA300 JE2 with the empty plasmid were assessed by RT-qPCR in (B1) Iron-depleted media (DIP = TSB + dipyridyl 400 μM). (B2) Iron-repleted media (DIP + 50 μM FeSO_4_). The bars represent the means of relative expression from at least three independent experiments. The error bars show standard errors of the means. Statistical differences were determined by Mann-Whitney U Test. (*P < 0.05; n.s. = not significant)

To determine if the Rsp activation acts in iron-independent manner, we measured the level of expression of *cntK*, *cntA* and *cntE* under varying metal conditions. As it is shown in Fig. 2a, the transcript levels of the three genes in the *rsp* mutant strain decreased in the three conditions tested, rich media (TSB), metal-depleted (TSB + DIP) and metal-replete medium (TSB + DIP + Fe) relative to the wild type strain (USA300 JE2). A 2-fold change was detected in TSB for all the genes of the operon. A slightly smaller decrease (1.9-, 1.7- and 1.5-fold changes for *cntK*, *cmtA* and *cntE*, respectively) was observed under DIP conditions. Additionally, the decrease was amplified with addition of 50 μM FeSO_4_ to DIP media (4.1-, 5.3- and 6.3-fold changes for *cntK*, *cntA* and *cntE* respectively) (Fig. 2a), likely due to the maximum level of repression of Fur in an iron-rich media. These data suggest that Rsp can activate the *cnt* operon under varying levels of iron-dependent repression by Fur, thereby supporting a basal level of the *cnt* gene expression in the cells. In complementation assays in the iron-depleted media, the decrease in *cnt* gene expression observed in the *rsp* mutant strain carrying the empty plasmid pTZN10 (3.3-, 2- and 3.2-fold changes detected for *cntK*, *cmtA* and *cntE*, respectively) was complemented to wild type levels with the plasmid containing *rsp* (pRsp). (1.4-, 0.92- and 1.2-fold changes for *cntK*, *cntA* and *cntE*, respectively) (Fig. 2B1). The same complementation was observed in the iron-repleted media, when pRsp was introduced into the *rsp* mutant. Compared to the *rsp* mutant strain with the empty plasmid pTZN10, the *rsp* strain with pRsp exhibited changes of 3.47- vs 1.35-fold for *cntK*, 3.3-vs 0.96-fold for *cntA* and 2.7-vs 0.9-fold for *cntE*, further confirming the effect of the Rsp activation in varying iron conditions (Fig. 2B2).

### Relationship between the repressors Fur and Zur and the activator Rsp in the regulation of the *cnt* operon

**Fig. 3:**
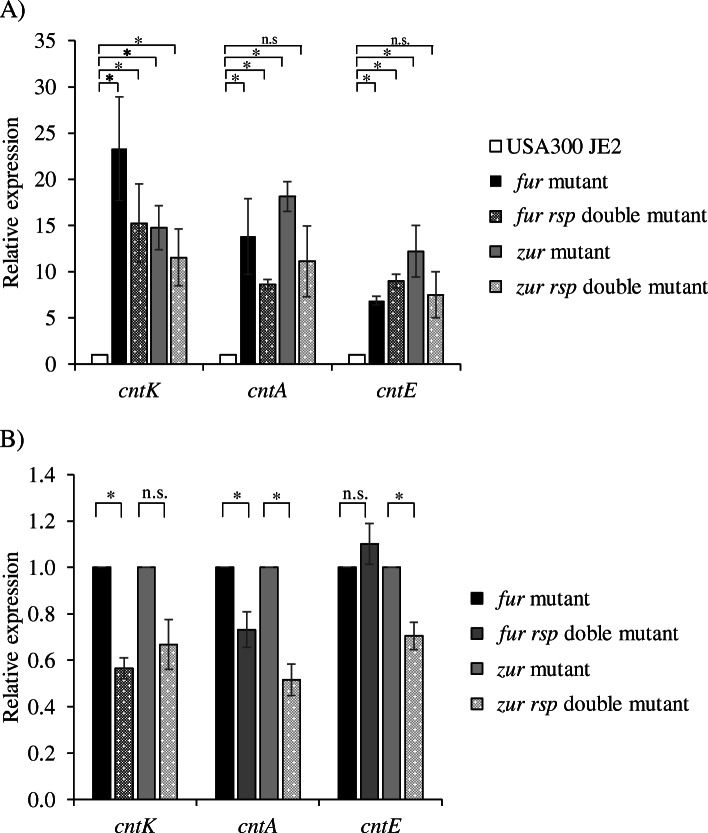
Rsp activation of the *cnt* operon can act independently of the repressors Fur and Zur. Relative expression of *cntK*, *cntA* and *cntE* genes in the *fur*, *zur* single mutants and *fur rsp, zur rsp* double mutants relative to USA300 JE2 strain in rich media (TSB) were assessed by RT-qPCR. The bars represent the means of relative expression from at least three independent experiments. The error bars show standard errors of the means. Statistical differences were determined by Mann-Whitney U Test except for the comparisons of the parental strain vs the *fur* mutant and the parental strain vs the *zur* mutant in which a unpaired Student’s t-test was performed (*P < 0.05)

As noted, Fur negatively regulates transcription of the *cnt* operon in an iron-dependent manner [8]. In addition, zinc regulation of the *cnt* operon through the repressor Zur has been previously reported [8]. To determine the possible interaction of Rsp with Fur and Zur in controlling *cnt* expression, we created single (*fur*, *zur*) and double mutants (*fur rsp*, *zur rsp*) in a USA300 background and measured the expression of *cnt* genes. As expected, the expression of *cntK*, *cntA* and *cntE* genes was increased substantially in the *fur* mutant (23-,14-, and 7-fold, respectively) as well as the *zur* mutant (15-, 17-, and 13-fold, respectively) (Fig. 3a). Although these increases were reduced in the *rsp* double mutants (by1.5- to 1.6-fold for *fur rsp* and by 1.3- to 1.6-fold for *zur rsp*) relative to single mutants there remained substantial increases in *cnt* gene expression relative to the parental strain the double mutants (7- to 15-fold for *fur rsp* and 7- to 12-fold for *zur rsp*). Thus, repression by Fur and Zur can occur in the presence and absence of Rsp activation, however, was sufficient to significantly partially counter Fur repression for *cntK* and *cntE* and to partially counter Zur repression for *cntA and cntE* (Fig. 3b).

### Binding of Rsp to the promoter region of the *cnt* operon

**Fig. 4:**
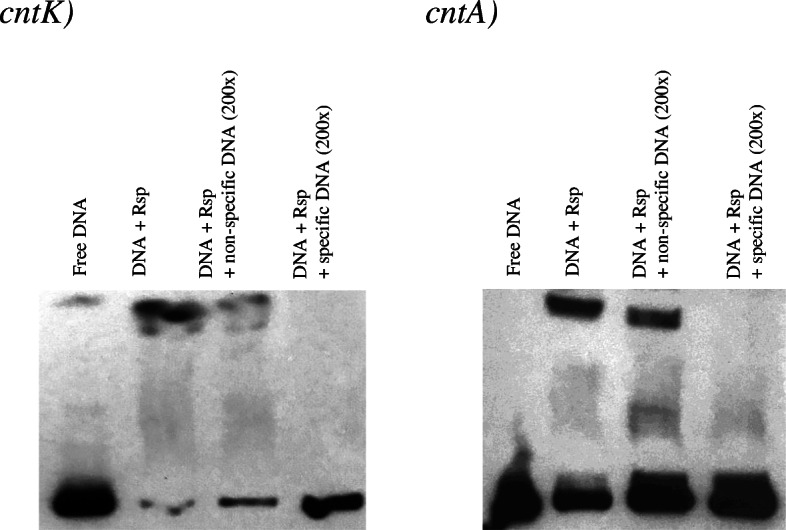
Gel mobility shift assay with purified Rsp protein mixed with the biotinylated 494-bp sequence upstream of *cntK* (*cntk*) and (*cntA*) 381-bp sequence upstream of *cntA.* Eight fmol of the biotin-labeled DNA was incubated with 1.5 μg of purified protein. The reaction mixture was incubated for 20 min at room temperature and analyzed by 5% nondenaturing polyacrylamide gel electrophoresis (PAGE). For the competition assay, a 200-fold excess of unlabeled specific DNA or nonspecific salmon sperm DNA was added to the reaction mixture prior to the incubation

To determine if Rsp acts directly to modulate expression of *cnt* genes, Rsp was expressed in a pQE-9 (His tag expression vector) previously constructed [11] using *E. coli* SG13009 containing the pREP4 plasmid as a host. After induction by IPTG and further purification on a nickel affinity column, an SDS-PAGE gel indicated a homogenous single protein band (data not shown). Two promoters have been previously determined for the *cnt* operon, one within the upstream region of *cntK* (*cntK* promoter) and the second one within the intergenic region between *cntM* and *cntA* genes (named *cntA* promoter) [8]. Incubation of Rsp with either the 494-bp *cntK* or the 381-bp *cntA* promoter fragments resulted in a DNA band shift (Fig. 4). These bands shifts were reduced in the presence of 200-fold excess unlabeled specific promoter region DNA and remained unchanged in the presence of a similar excess of salmon sperm DNA, indicating specific binding to the promoter DNA fragment.

## Discussion

The Cnt system is essential for the optimal import of nickel, cobalt, zinc and ferrous iron when these metals are limited, and it contributes to virulence in *S. aureus* [6, 12, 13].

Several studies have identified Fur and Zur as repressors of the Cnt system [6–8] but, no activators of the operon have been identified. In this study, we identified that the AraC-type global regulator Rsp is involved in the activation of the *cnt* operon by binding directly to the *cntK* and *cntA* promoters. Since both a Fur box and a Zur box are present upstream of *cntK* and *cntA* promoters [8], it is possible that Rsp binding involves one of those boxes. However, the expression of the Fur-regulated *sbn* operon [14], was not changed in a *rsp* mutant (data not shown), suggesting that Rsp does not bind to the Fur boxes in general. Another study demonstrated that Rsp upregulates *agrA* and downregulates *ica* by direct binding of their promoters [11]. The predicted binding motifs of the *agrP2* and the *ica* promoters, however, are different [11] and at the same time no similar sequence was found in the *fnbA* promoter, which reportedly also binds Rsp [15]. Those possible binding motifs were also not present in the *cnt* promoters. These findings favor our hypothesis that Rsp does not bind to the Fur box, and determination of the binding motif of Rsp will await further study. The upregulation of the Cnt system by Rsp was demonstrated to occur in both low and high metal conditions suggesting that Rsp acts to maintain a basal level expression of the *cnt* operon in the presence of Fur-mediated iron-dependent repression. Notably the effect was greatest when Fur repression was increased under high iron conditions. To evaluate the interaction of Rsp with Fur or Zur repressors, we constructed double mutants. Notably the increases in *cnt* gene expression levels in *fur* and *zur* single were not eliminated in the *rsp* double mutants. A partial counter effect of Rsp on Fur repression was seen for *cntK* and *cntE* and a partial counter effect of Rsp on Zur was seen for *cntA* and *cntE*. Thus, Rsp, Fur, and Zur can act independently of each other in affecting levels of *cnt* gene expression.

## Conclusions

In conclusion, we identified for the first time an activator of the Cnt system, Rsp, that acts in both low and high iron conditions and contributes to a basal level of production of the StP synthesis genes and StP importers to scavenge free metals when needed. Rsp acts in addition to the known regulation by repressors Fur and Zur. This tight and complex regulation also could prevent the toxicity due to the StP accumulation by the activation of the expression of the StP exporter *cntE* gene [16].

## Methods

### Bacterial strains and growth conditions

All the strains and plasmids used in this study are summarized in Table 2. *S. aureus* strains were routinely cultivated in trypticase soy broth (TSB) and on tryptic soy agar plates (TSA), while *E. coli* strains were grown in Luria-Bertani broth (LB). Bacteria were grown at 37 °C unless stated otherwise. The following antibiotics were obtained from Sigma-Aldrich (St. Louis, MO) and used for plasmid maintenance at the concentrations indicated: ampicillin, 100 μg/ml; kanamycin, 25 μg/ml or 75 μg/ml; and tetracycline, 10 μg/ml.

**Table 2:** The strains and plasmids used in this study

Strains or plasmids	Genotype or characteristic (s)^a^	References
***S. aureus*** **strains**		
USA300 JE2	Parental strain for the Nebraska Transposon Mutant Library	[10]
NE1304	*rsp* mutant from the Nebraska Transposon Mutant Library with *erm* gene insertion; Erm ^R^	[10]
Newman *fur::tet*	*fur* mutant with *tet* gene insertion, Tet^R^	[12]
NCTC8325 *zur::tet*	*zur* mutant with *tet* gene insertion, Tet^R^	[13]
USA300 *fur*	*fur* mutant with *tet* gene insertion, Tet^R^	This study
USA300 *zur*	*zur* mutant with *tet* gene insertion, Tet^R^	This study
USA300 *fur rsp*	*fur rsp* double mutant; Tet^R^, Erm^R^	This study
USA300 *zur rsp*	*zur rsp* double mutant; Tet^R^, Erm^R^	This study
***E. coli*** **strains**		
SG13009 pREP4 (pQE-9-*rsp*)	Derived from *E. coli* K12 harboring a plasmid to overexpressed *rsp.*	[11]
**Plasmids**		
pZTN10	*Escherichia coli*/*Staphylococcus aureus* shuttle vector; Amp^R^, Cm^R^	[17]
pTZN10-rsp (pRsp)	pTZN10 derivative containing *rsp* gene from *S. aureus;* Amp^R^, Cm^R^	This study

^a^*Abbreviations*: *Erm* ^*R*^ Erythromycin resistance, *Tet*^*R*^ Tetracycline resisatnce, *Amp*^*R*^ Ampicillin resisatnce, *Cm*^*R*^ Chloramphenicol resistance

### Construction of *S. aureus* mutant strains

*S. aureus* USA300 *fur* and USA300 *zur* single mutants and USA300 *fur rsp* and USA300 *zur rsp* double mutants strains were created via bacterial phage φ85 transduction from *S. aureus* Newman *fur::tet* and *S. aureus* NCTC8325 *zur::tet* strains respectively as previously described [5]. Colonies of interest were selected on TSA plates containing sodium citrate (10 μg/ml) and tetracycline (10 μg/ml). Mutant candidates were isolated, and the mutants were confirmed by PCR and DNA sequencing.

### Construction of Rsp complementation plasmid

To complement the *rsp* mutant, the entire *rsp* gene was amplified from USA300 chromosomal DNA using primers listed in supplementary table (Table S1) and cloned into shuttle vector pTZN10 [17]. The plasmid was extracted from *E. coli* DH5α, introduced into *S. aureus* RN4220, then into USA300 mutants, and maintained by the addition of chloramphenicol (10 μg/ml) to the culture medium. The plasmid insert was verified by DNA sequencing.

### RNA extraction and relative expression of the *cnt* operon genes by RT-qPCR

Cultures were inoculated from − 80 °C stocks into TSB broth supplemented with the appropriate antibiotic, grown at 37 °C overnight, diluted 1:100 into fresh TSB broth with the same antibiotics, and grown for 2 to 3 h to an optical density at 600 nm (OD_600_) of ~ 0.5. To measure *cnt* operon expression under iron-depleted medium, 400 μM dipyridyl (DIP) was added at an OD_600_ of ~ 0.5 with or without the supplementation of 50 μM FeSO_4._ RNA Protect Bacteria Reagent (Qiagen) was added to the culture for immediate RNA stabilization prior to harvest the cells. Total *S. aureus* RNA was isolated using the Qiagen RNeasy mini kit (Qiagen, Valencia, CA) following the manufacturer’s instructions and using lysostaphin for cell lysis. Reverse transcription followed by real-time quantitative PCR (RT-qPCR) was performed to determine the expression levels of the *cnt* operon genes. Comparison was made to the expression level of the housekeeping gene *gmk* [5]. cDNA was generated by reverse transcription using the Verso cDNA synthesis kit (Thermo Scientific) according to the manufacturer’s protocol. SsoFast EvaGreen supermix and the QuantStudio 3 Real-time PCR system (Thermo Scientific) were employed for RT-qPCR using the synthesized cDNA as the template. Primers used are listed in the supplementary table (Table S1). At least three different assays with three independent cultures and RNA extractions were performed for each gene tested. Two-group comparisons of gene expression levels were evaluated by the Mann-Whitney test with the exception of the Students’s t-test used for comparison of parental and *fur* and *zur* single mutants.

### Expression and purification of histidine-tagged Rsp protein

*E. coli* SG13009 (pREP4) cells harboring *rsp* cloned into pQE-9 vector [11] were grown in LB supplemented with ampicillin 100 μg/ml plus kanamycin 25 μg/ml at 37 °C (OD_600_ of ~ 1) and then subjected to induction by 0.5 mM isopropyl-β-d-thiogalactopyranoside (IPTG) for 4 h. After centrifugation at 10,000 rpm for 30 min at 4 °C the cells were resuspended in lysis buffer (50 mM NaH_2_PO_4_, pH 8, 300 mM NaCl) at 0.04 volume of the original culture. We added benzonase nuclease (Sigma-Aldrich) and a complete mini protease inhibitor cocktail tablet (Roche) to the lysate for further sonication on ice for 10 cycles of 30 s with 30 s resting periods followed by centrifugation at 10,000 rpm for 30 min at 4 °C and filtering the supernatant through a Steriflip-GP, 0.22-μm, polyethersulfone membrane (EMD Millipore, Billerica, MA). Nickel affinity chromatography (GE Healthcare, Marlborough, MA) using 5 ml fractions and incremental concentrations of imidazole was performed for purification of the histidine-tagged Rsp protein. The fractions obtained were run on SDS-PAGE Coomassie gels and Western blotting (Invitrogen, Carlsbad, CA) was performed following the manufacturer’s protocols. The appropriate fractions were then cleared of imidazole using PD-10 desalting columns (GE Healthcare, Marlborough, MA) and concentrated on Amicon Ultra-15 (Ultracel 50 K) centrifugal filter units (EMD Millipore). The concentration was measured using a NanoDrop 1000 spectrophotometer (Thermo Scientific).

### Electrophoretic DNA mobility shift assays

Using a LightShift chemiluminescent EMSA kit (Thermo Scientific, Wilmington, DE), the binding of Rsp to the labeled 494-bp region upstream of *cntK* and 381-bp region upstream of *cntA* was assessed. Both *cntK* and *cntA* fragments were amplified using a biotinylated 5′ primer (Integrated DNA Technologies, Coralville, IA) (Table S1) and diluted to ∼8 fmol per reaction. LightShift chemiluminescent EMSA kit controls were run alongside mixtures of biotinylated DNA and various protein concentrations (65 to 365 μg/ml) on an acrylamide nondenaturing gel, transferred to a 0.45-μm nylon membrane (Thermo Scientific, Wilmington, DE), and detected on autoradiography film (GE Healthcare, Marlborough, MA). Specificity of binding was determined by addition of 200-fold excess of specific unlabeled DNA in comparison to the same excess of nonspecific salmon sperm DNA.

## Supplementary information

**Additional file 1 **: **Table S1** Primers used in this study.

## Data Availability

All data generated or analyzed during this study are included in this published article [and its supplementary information files]. USA300 *fur* and USA300 *zur* mutants have been included in the GenBank with accession numbers MW048529 and MW048528 respectively.

## Abbreviations

Stp: Staphylopine
TSB: Trypticase soy broth
TSA: Tryptic soy agar plates
LB: Luria-Bertani broth
RT-qPCR: Real-time quantitative PCR
DIP: Dipyridyl
IPTG: Isopropyl-β-d-thiogalactopyranoside
SEM: Error standard of the mean
Erm ^R^: Erythromycin resistance
Tet^R^: Tetracycline resisatnce
Amp^R^: Ampicillin resisatnce
Cm^R^: Chloramphenicol resistance
