# Supplementary material for: Rsp activates expression of the Cnt system in Staphylococcus aureus
Source: BMC Microbiol. 2020 Oct 28;20:327. doi: 10.1186/s12866-020-02013-0 (PMC7594338; doi:10.1186/s12866-020-02013-0)
Supplement: Supplementary file 1 — Additional file 1 : Table S1 Primers used in this study. [file 12866_2020_2013_MOESM1_ESM.docx]

**Table S1** Primers used in this study.

| **Name^a^** | **Sequence 5'-3'^b^** | **Size (bp)** | **References** |
| --- | --- | --- | --- |
| **PCR primers** |  |  |  |
| Fur-F | GGACATCGTTGGAAGAACG | 466 | [1] |
| Fur-R | GCAATTTACTATCCTTTACC |  |  |
| Zur-F | GCGAATAACAAAAAGTTTTAAGCA | 724 | This study |
| Zur-R | TTGCCATAAAATCATACGAACG |  |  |
| Rsp-F | ATGACATGCCAACTTAAAAT | 2106 | This study |
| Rsp-R | TTAGCTTGGTTTAAAGCAAAT |  |  |
| Rsp-compF-KpnI | GCTA**GGTACC**ATGACATGCCAACTTAAAATA | 2106 | This study |
| Rsp-compR-SacI | GCTA**GAGCTC**TTAGCTTGGTTTAAAGCAA |  |  |
| **RT-qPCR primers** |  |  |  |
| RTcntK-F | CACGCACAAACGCTTCAAC | 160 | This study |
| RTcntK-R | CAAATGCCACAAGCCCATC |  |  |
| RTcntL-F | CCCTAGGTCAACGGCTTGT | 198 | This study |
| RTcntL-R | CAGGCGTCTACCACAGGTTACT |  |  |
| RTcntM-F | CTGACGGCACTCTAAATGCTTG | 188 | This study |
| RTcntM-R | GCTTTATGTGCACCCACCAC |  |  |
| RTcntA-F | AGCGCCTGAACGCTCTTTAC | 204 | This study |
| RTcntA-R | GCTGAACGTCGTACTTCTGGTG |  |  |
| RTcntB-F | GATTTCACACCGCTTGCTCT | 159 | This study |
| RTcntB-R | GCCGACTTCTGGATTAACAGG |  |  |
| RTcntC-F | CAGTAGGCGCTTTGACACCT | 232 | This study |
| RTcntC-R | CGTTGGGCATGGTTCTGT |  |  |
| RTcntD-F | TCGCTGTAACATTCCTCCTG | 185 | This study |
| RTcntD-R | GTGCCTTTGACCCATCAACT |  |  |
| RTcntF-F | AGACCGACTTCTTCCAAC | 265 | This study |
| RTcntF-R | GTGGTAGCGGTAAATCGACA |  |  |
| RTnorD-F (cntE) | ATGAAAGGTGCAATGGCT | 101 | [2] |
| RTnorD-R (cntE) | CCTCGTAAAGGTATAAA |  |  |
| gmk-qPCR-F | TCAGGACCATCTGGAGTAGGTAAAG | 108 | [2] |
| gmk-qPCR-R | TTCACGCATTTGACGTGTTG |  |  |
| **EMSA primers** |  |  |  |
| PcntA-F | CTAAACCTTTATTACCGCCAC | 381 | [3] |
| PcntA-Biotin-F | Biotin-CTAAACCTTTATTACCGCCAC | 381 |  |
| PcntA-R | GATTGATCAGTTCCTAACTCGC |  |  |
| PcntK-F | ACTTAGTAATCGGTTGTTGTATC | 494 | [3] |
| PcntK-Biotin-F | Biotin-ACTTAGTAATCGGTTGTTGTATC | 494 |  |
| PcntK-R | CAACTTGACGATGAAAATTCG |  |  |

^a^ F, forward primer; R, reverse primer.

^b^ Bold indicates restriction sites.

**References**

1. Torres VJ, Attia AS, Mason WJ, Hood MI, Corbin BD, Beasley FC, Anderson KL, Stauff DL, McDonald WH, Zimmerman LJ, Friedman DB, Heinrichs DE, Dunman PM, Skaar EP. *Staphylococcus aureus* *fur* regulates the expression of virulence factors that contribute to the pathogenesis of pneumonia. Infect Immun. 2010; 78:1618-28.
2. Truong-Bolduc QC, Hsing LC, Villet R, Bolduc GR, Estabrooks Z, Taguezem GF, Hooper DC. Reduced aeration affects the expression of the NorB efflux pump of *Staphylococcus aureus* by posttranslational modification of MgrA. J Bacteriol.2012; 194:1823-34.
3. Fojcik C, Arnoux P, Ouerdane L, Aigle M, Alfonsi L, Borezée-Durant E. Independent and cooperative regulation of staphylopine biosynthesis and trafficking by Fur and Zur. Mol Microbiol. 2018; 108:159-77.
